# Post Ambulatory Swollen Hands (POTASH): An Autobiographical Case Report

**DOI:** 10.7759/cureus.19312

**Published:** 2021-11-06

**Authors:** Philip R Cohen

**Affiliations:** 1 Dermatology, University of California, Davis Medical Center, Sacramento, USA

**Keywords:** swollen, swelling, sign, running, palm, hand, fist, edema, autobiographical case report, ambulatory

## Abstract

Post ambulatory swollen hands (POTASH) is a rarely described etiology for hand swelling; to the best of my knowledge, it has only been reported in the medical literature a decade ago in a prospective study evaluating its development in walkers who were either dog owners (who walked or did not walk their dogs) in comparison to non-dog owners. In addition to swelling after initiating ambulation by participating in an activity such as hiking, running, or walking, there are also several other causes of swollen hands; a positive fist sign has only been described in a limited number of conditions observed in individuals with hand swelling. A fist is created when there is clenching of the fingers and the fingertips are in direct contact with the palm of the hand with the thumb lying on top of the fingers between the proximal and distal interphalangeal joints. A positive fist sign is demonstrated by the inability to clench the fingers tightly into a fist; indeed, it is a common--yet not frequently reported--manifestation observed in individuals with a swollen hand. In contrast, a negative fist sign occurs when the patient can form a fist of tightly clenched fingers. The author, a 62-year-old physician and long-distance runner since high school, developed recurrent episodes of POTASH beginning five years ago. He noticed asymptomatic, bilateral, and symmetric swelling of his dorsal and palmar hands--with a positive fist sign--beginning after approximately one hour of running; the degree of swelling was proportional to the duration of time he ran. His hand swelling would completely resolve spontaneously--and his fist sign would be negative--within two hours after he stopped running. Recommendations for hikers and walkers to potentially eliminate or limit the degree of POTASH have been suggested; for dog owners who walked their dog, POTASH was less likely to occur if they regularly walked the dog. Several etiologies for POTASH have been proposed; however, the definitive pathogenesis for hand swelling related to either hiking, running, or walking remains to be determined. Therefore, research to gain additional insight and possibly establish the cause of ambulatory-associated swollen hands is warranted.

## Introduction

Hand swelling can be unilateral or bilateral. The onset of the swelling can be acute or chronic. In addition, swelling of the hands can be asymptomatic or associated with numbness, pain, pruritus, tingling, or warmth [1-3].

The potential etiology of swollen hands is diverse, and the extent of involvement is variable. Hand swelling can result from either intrinsic (such as a tumor) or extrinsic (such as an arthropod assault) causes. The swelling can be limited to only the hands (such as allergic contact dermatitis) or a component of a systemic condition (such as mixed connective tissue disease) [1-20].

The author, a 62-year-old male physician, developed recurrent episodes of swollen hands, including the digits. This would occur during long distance running, such as half marathon competitions; the hand swelling would spontaneously resolve within a few hours after he stopped running. The features and pathogenesis of post ambulatory swollen hands (POTASH) are described, and the differential diagnosis of acute and chronic hand swelling is summarized.

## Case presentation

A 62-year-old man repeatedly developed asymptomatic swelling of his hands. This acquired condition began approximately three to five years earlier. It was associated with exercise--specifically running. It never occurred during any other activities, including those of daily living.

Typically, the swollen hands only occurred while he was running long distances of greater than four miles. Indeed, he particularly noticed a morphologic change of both hands while participating in half marathons. The swelling would often begin after he had completed about one hour of running--which corresponded to the onset of the hand changes occurring after about four miles of the 13.1 miles race.

The swelling only occurred on his hands. There is no enlargement of the arms or the forearms proximal to the wrists. Also, neither his feet, face (such as his eyelids) nor mucous membranes (such as lips) developed swelling.

The current episode of hand swelling occurred during April 2021, in San Diego, California, United States of America; the ambient temperature was 57 degrees Fahrenheit. However, his running-associated swollen hands has also occurred during running races during the summer when the outside temperature ranged from 80 degrees to the mid 90 degrees Fahrenheit. In addition, the condition has manifested during similar races in cooler climates when the temperature at the beginning of the race was in the upper 30 degrees Fahrenheit and only peaked to the mid or upper 40 degrees Fahrenheit. 

In the present case, he began running at 11:00 am; the overlying sky was overcast with clouds and there was a cool breeze. He was not wearing mittens or gloves on his hands. Painless swelling of both hands, including all their digits, was present by 12:00 pm. However, there was no pruritus, and no additional areas of his body were swollen. 

**Figure 1:**
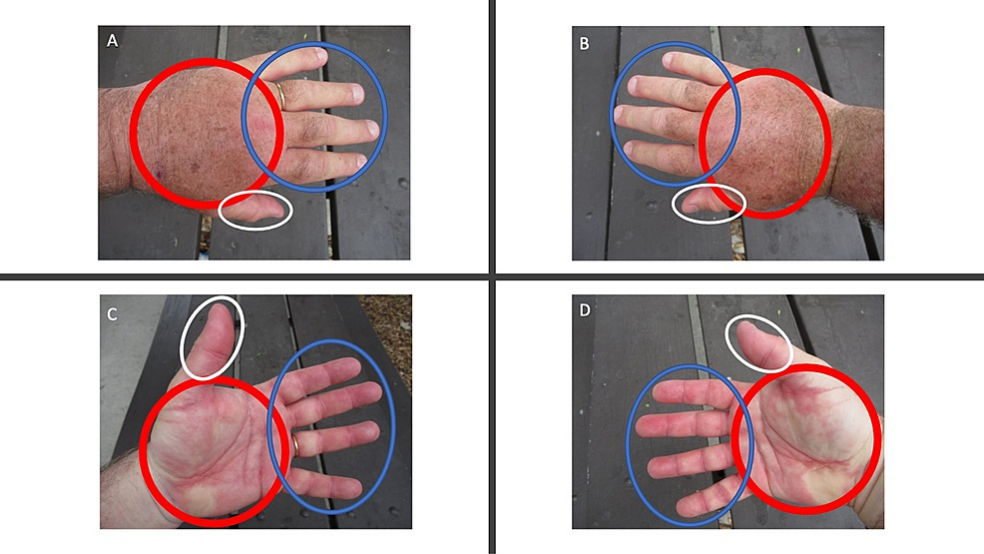
Post ambulatory swollen hands (POTASH) that developed during a half marathon A 62-year-old man presented with a swelling of not only the dorsal hands (red ovals), fingers (blue ovals), and thumbs (white ovals) (A and B), but also the palms (red ovals), ventral thumbs (white ovals) and fingers (blue ovals) (C and D). There is swelling proximal and distal to the gold wedding band on his left fourth finger (A and C). The edema is prominent and the skin lines, superficial vessels, and tendons on the dorsal hands (red ovals) cannot be seen (A and B). The ventral swollen thumbs (white ovals) and fingers (blue ovals) show erythema of the digits; there is blanching of the edematous palms (red ovals) (C and D).

He completed the race after three hours and 44 minutes. Cutaneous examination showed pronounced swelling of both hands and all accompanying digits (Figure 1). The swollen dorsal hands revealed a loss of skin lines, superficial blood vessels, and tendons. In addition, there was swelling both proximal and distal to the ring on the fourth finger of his left hand. The palms were also both swollen; there was blanching of the skin. All the ventral fingers and thumbs were not only swollen but also erythematous.

**Figure 2:**
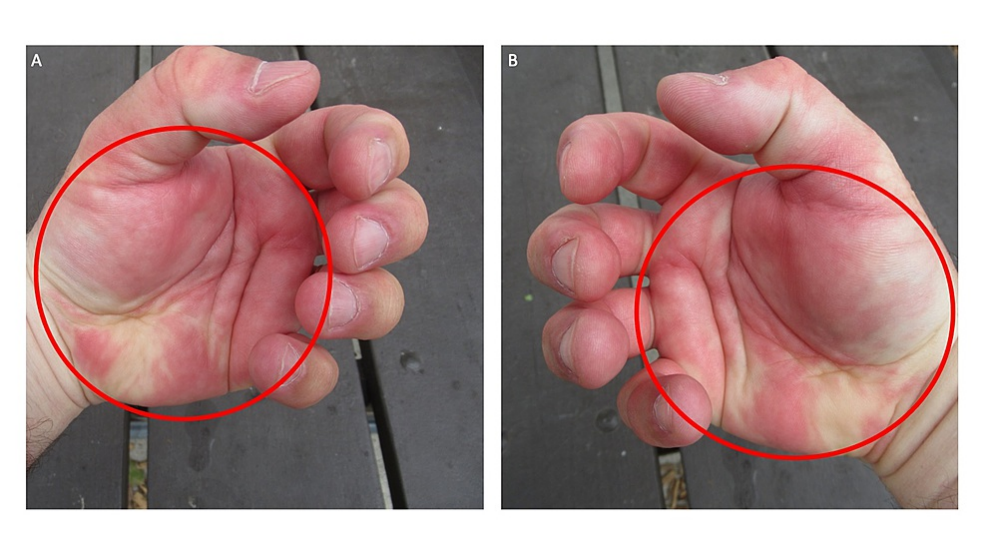
Post ambulatory swollen hands (POTASH)-associated positive fist sign The fist sign is positive when an individual is not able to make a fist; therefore, when the person attempts to clench their fingers, the fingertips are not able to contact and be hidden by the ipsilateral palm. The left (A) and right (B) edematous swollen palms show erythema and focal blanching (red ovals) when the patient attempts to clench his finger in a futile attempt to make a fist. The positive fist sign associated with POTASH demonstrates the limited range of finger motion during a maximal attempt to clench the fingers into the palm.

He was not able to make a fist (Figure 2). He achieved very limited movement when he attempted to clench his swollen fingers toward the palms. The edematous palms were both blanched and erythematous. The inability of fingers to be able to contact the ipsilateral palm when an individual is trying to make a fist is referred to as a ‘positive’ fist sign; he had bilateral positive fist sign.

**Figure 3:**
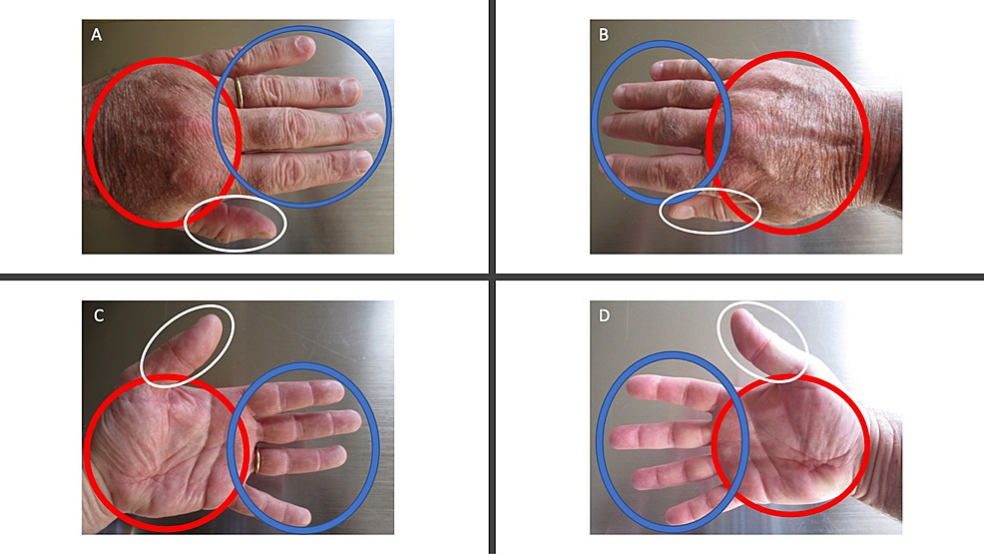
Complete spontaneous resolution of running-associated post ambulatory swollen hands (POTASH) All the hand and digital swelling associated with POTASH completely resolved spontaneously within two hours after the patient stopped running. There is no edema in the previously swollen dorsal left (A) and right (B) hands (red ovals); skin lines, superficial blood vessels, and tendons can be seen. Previous swelling of the dorsal thumbs (white ovals) and fingers (blue ovals) is gone (A and B). Dorsal (A) and palmar (C) views of the fourth finger do not show any swelling proximal or distal to the gold ring (blue oval). The creases on the palms (red ovals) are deeper and more prominent (C and D); there is no swelling of the ventral thumbs (white ovals) and fingers (blue ovals) (C and D). Photographs of the hands were taken 24 hours after the patient stopped running.

Within two hours after he had stopped running, the swelling of both hands and all digits had resolved. Cutaneous examination the following day, approximately 24 hours after he had stopped running, showed complete spontaneous resolution of the swelling (Figure 3). Skin lines, superficial vessels, and tendons on the dorsal hands were apparent; there was no swelling proximal or distal to the ring on his left fourth finger. His palmar creases were more prominent and there was neither blanching nor erythema.

**Figure 4:**
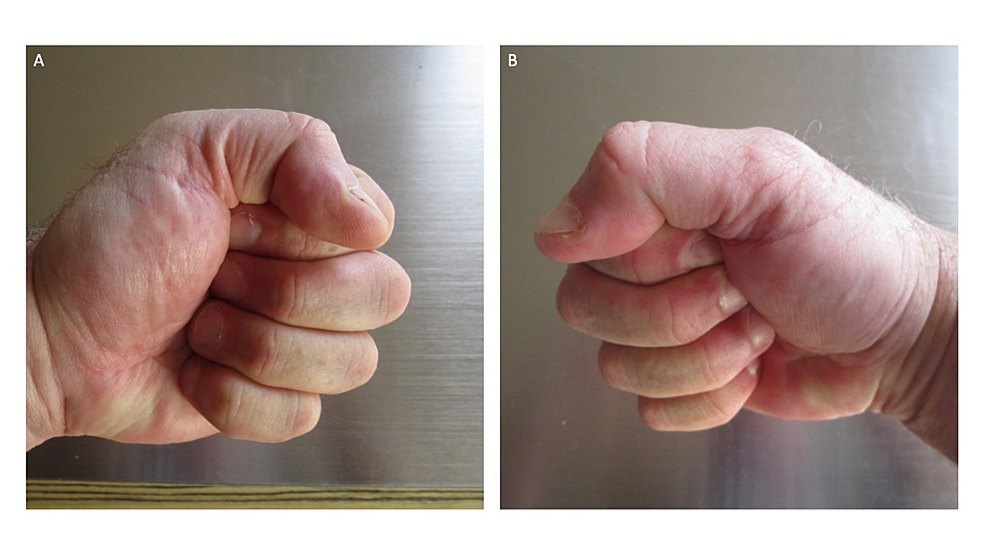
Bilateral negative fist sign after post ambulatory swollen hands (POTASH), which had been associated with running, has spontaneously resolved completely A negative fist sign is observed when the individual can make a tight fist after clenching the fingers into the ipsilateral palm. Within two hours after the patient stopped running, he could make a fist with his left (A) and right (B) hands. Photographs of the hands were taken 24 hours after the patient stopped running.

He was now able to make a tight fist (Figure 4). His fingers easily contacted the ipsilateral palm, and the fingertips were nearly hidden. He now had a ‘negative’ fist sign.

## Discussion

The unanticipated development of hand swelling may be a cause of concern and often requires additional assessment [3]. The initial evaluation should determine whether the onset of hand swelling was acute (for which some of the causes are listed in Table 1 [1-11]) or chronic (for which some of the causes are listed in Table 2 [3,12-20]). In addition, the examination should consider the presence of numbness, pain, pruritus, tingling or warmth (or the absence of symptoms), the color changes of the affected area, and whether the condition persists, or resolves (either spontaneously or following treatment). The acute presentation of painful or numb or warm, unilateral or bilateral, hand swelling with accompanying skin color changes (suggestive of infection or thromboembolic phenomenon) or trauma to the affected area may necessitate urgent medical attention [3,5].

**Table 1:** Differential diagnosis of acute hand swelling COVID-19: coronavirus disease 2019; CR: current report; POTASH: post ambulatory swollen hands; Refs: references; RS3PE: remitting seronegative symmetrical synovitis with pitting edema; SARS-CoV-2: severe acute respiratory syndrome coronavirus 2

Diagnosis	Comments	Refs
Allergic contact dermatitis	Painful and pruritic, progressive erythema and bilateral hand swelling of two days duration in a 45-year-old man. He began using a new antiseptic hand wash during the prior seven days. Patch testing confirmed that the contact allergen was the antiseptic chloroxylenol.	Dickson and Fischer, 2019 [4]
Compartment syndrome	There are several etiologies for compartment syndrome: bites (crotalid snake envenomation and arthropod assault from spiders and scorpion), bleeding (hypercoagulable state and vascular injury), burns (electric and thermal), constrictive cast or bandages with prolonged traction, high-pressure injection, infection (abscess and necrotizing fasciitis), infiltrated intravenous line or infusion, muscle overuse (exercise, seizure, and tetany), reperfusion injury, and trauma (crush injuries, direct muscular contusion, fractures, and prolonged limb compression. In addition to hand swelling, physical findings (which include the five Ps) are disproportionate pain, pallor, paralysis, paresthesia, and pulselessness. To preserve hand function and avoid tissue damage, early recognition is crucial and compartment release (utilizing emergency fasciotomy) may be necessary.	Oak and Abrams [5]
Deep venous thrombosis	A thrombus of a deep upper extremity vein can present with painful swelling of the arm and hand; warmth and erythema or discoloration are also present. It can be caused by damage to the blood vessel walls (such as from venous catheter placement), hypercoagulability, and/or stasis.	Studdiford and Stonehouse, 2009 [3]
Erythromelalgia	Primary or secondary erythromelalgia presents with recurrent episodes of bilateral and symmetrical, erythematous, warm, painful (such as burning) swollen hands and feet. Relief results from elevation of and cold exposure to the extremity. Low-dose daily aspirin can provide resolution of primary erythromelalgia. Secondary erythromelalgia can be associated with multiple conditions including myeloproliferative disorders.	Hart, 1996 [6]
Exercise-induced urticaria	Exercise-induced urticaria is part of a range of disorders which includes exercise-induced anaphylaxis and food-dependent exercise-induced anaphylaxis. Hand swelling (presenting as hives) may be a component of exercise-induced urticaria; however, lesions are usually widespread. In addition to urticaria and collapse during or after exercise, other symptoms include angioedema, flushing, gastrointestinal symptoms, hypotension, pruritus, and respiratory symptoms.	Studdiford and Stonehouse, 2009 [3]
Infection	Cellulitis secondary to Staphylococcus aureus or Streptococcus pyogenes (erysipelas) can result in erythematous swelling of the hand.	Dickson and Fischer, 2019 [4]
Irritant contact dermatitis	Excessive hand washing can result in xerosis, fissures, and tender, potentially swollen, hands; the thinner skin of the dorsal hands is most susceptible than the thicker palmar skin.	Dickson and Fischer, 2019 [4]
Medications	Medication-induced edema may occur immediately or up to several weeks after starting a drug. It may be generalized or restricted to specific areas such as swollen lower extremities or hand swelling. Usually, it resolved within days after stopping the causative agent. Medications commonly associated with edema include antidepressants (monoamine oxidase inhibitors, tricyclics, and trazodone), antihypertensives (beta-adrenergic blockers, calcium channel blockers, clonidine, hydralazine, methyldopa, and minoxidil), anti-Parkinsonism drugs (pramipexole), antivirals (acyclovir), chemotherapeutics (cyclosphosphamide, cytosine arabinoside, and mithramycin), cytokines (granulocyte colony-stimulating factor, granulocyte-macrophage colony-stimulating factor, interferon alpha, and interleukin-2), hormones (androgens, corticosteroids, estrogen, progesterone, and testosterone), hypoglycemic agents (thiazolidinediones: pioglitazone and rosiglitazone), immunosuppressants (cyclosporine), and nonsteroidal anti-inflammatory drugs (celecoxib, ibuprofen, and naproxen).	Studdiford and Stonehouse, 2009 [3]
Nocturnal hand swelling	In healthy individuals without active or prior hand pathology, overnight (which was defined to be from 8 PM to 8 AM), there was an overall 4.5 percent increase in average hand volume. The consistent physiological nocturnal hand swelling was attributed to fluid retention; the hand volume returned to baseline during the next day (from 8 AM to 8 PM).	Warrender et al., 2019 [7]
POTASH	Post ambulatory refers to the onset of the asymptomatic hand swelling occurring after the initiation of--yet while still participating in--either hiking, running, or walking. Both the dorsum and palm of the hand become swollen; there is a positive fist sign: the individual cannot clench their fingers to make a fist. The hand swelling resolves spontaneously, usually within one to two hours, after the ambulatory activity has been discontinued.	Ravaglia et al., 2011 [1], DesMarais, 2021 [2], CR
Post COVID-19 puffy hands	Two women (28-years-old and 33-years-old) presented with non-resolving, isolated, bilateral, erythematous, tender to palpation, non-pitting edema of the hands and fingers with fissures over the interphalangeal joints. Six and eight weeks prior to the onset of their hand symptoms, respectively, each had a positive result for SARS-CoV-2 with real-time polymerase chain reaction testing of a nasopharyngeal swab sample. Hand swelling occurred after all acute COVID-19 viral symptoms had resolved and repeat testing for the virus was negative. The investigators proposed a capillary permeability hypothesis of COVID-19-related microvascular damage and acral capillary dysfunction with leakage resulting in puffy hands.	Ciaffi et al., 2021 [8]
Raynaud’s disease and phenomenon	Painful, red-purple to blue discoloration with swelling--that predominantly affects the distal fingers of the hands--occurs.	Ravaglia et al., 2011 [1], Bickel et al., 2017 [9]
RS3PE	This condition occurs in elderly individuals (over 50 years of age) with the sudden onset of seronegative symmetric polyarthritis and bilateral hand swelling (caused by pitting edema), with or without tenderness. The feet are also usually affected, and the rheumatoid factor is negative. Individuals have a positive fist sign; finger flexion is limited. The clinical features respond dramatically and rapidly to treatment with systemic corticosteroids.	Joshi et al., 2009 [10]
Thoracic outlet syndrome	There are several etiologies for thoracic outlet syndrome; a cervical rib is the most common. Venous thoracic outlet syndrome can result from effort-induced (such as strenuous and repetitive exercise of the upper extremities) thrombosis of the axillary and subclavian veins associated with compression of the subclavian vein between the clavicle and the first rib. Neurologic and vascular symptoms result from the compression of the subclavian artery or vein or both and/or the brachial plexus lower roots. In addition to the neurologic and vascular manifestations, pain, erythema or bluish discoloration, and hand swelling may also be observed. Management includes either conservative measures (such as analgesics and physical therapy), or a muscle block (using either a local anesthetic or botulinum toxin), or decompression surgery.	Ravaglia et al., 2011 [1], Studdiford and Stonehouse, 2009 [3], Kalchiem-Dekel et al., 2015 [11]

**Table 2:** Differential diagnosis of chronic hand swelling Refs: references

Diagnosis	Comments	Refs
Acromegaly	Acromegaly is a chronic disease characterized by excess growth hormone secretion by the pituitary gland and increased production of insulin-like growth factor-1 by the liver. Hands and feet become swollen and remain permanently enlarged. The patients have a positive fist sign: when they attempt to make a tight fist, they are not able to cover their fingernails with the center of the palm.	Harada et al., 2019 [12]
Carpal tunnel syndrome-tumor associated	Giant lipoma (characterized by lesions measuring five or more centimeters along the longest axis) are an uncommon (less than five percent of benign hand tumors) occurrence on the palm presenting as a swollen hand. Symptoms--including carpal tunnel syndrome--may occur from compression of the median nerve and include pain (burning), paresthesia (tingling), and loss of power (diminished grip). Patients have a positive fist sign which resolves after surgical removal of the lipoma; hand grip strength may also improve. Fibrolipomatous hamartoma of the median nerve and anomalies of the flexor digitorum superficialis muscle can also present with carpal tunnel syndrome and painful swelling of the palm.	Jalan et al., 2011 [13], Ranjan et al, 2021 [14]
Complex regional pain syndrome type I	This condition is also referred to as algodystrophy, reflex sympathetic dysytrophy, and Sudeck’s atrophy. In contrast to complex regional pain syndrome type II, complex regional pain syndrome type I (which is more common) has no major nerve lesion. Although a precipitating factor may not be identified, complex regional pain syndrome type I is usually associated with nerve injury caused by trauma or surgery. In addition to limb pain, dystrophic changes, motor changes, sensory changes, sudomotor changes, and vasomotor changes typically occur. Ipsilateral painful hand swelling may be noted, particularly after surgical procedures such as pacemaker implantation or arthroscopic rotator cuff repair. Treatment options include non-pharmacologic management (such as physical therapy, transcutaneous electrical nerve stimulation, desensitization, and sensory re-education of the limb), and drug therapy (such as adrenergic compounds, analgesics, anticonvulsants, antidepressants, bisphosphonates, calcium channel blockers, corticosteroids, membrane-stabilizing agents, and neurotropin).	Kamath and Rao, 2015 [15]
Leprosy (Hansen’s disease)	A 24-year-old woman presented with facial and painful bilateral hand swelling of one month’s duration. She also had tender nodules on her arms and legs; biopsy of an arm lesion showed a lobular panniculitis with overlying acute and chronic dermal inflammation. Acid-fast and Fite stains revealed bacilli within histiocytes and cutaneous nerves. She was diagnosed with lepromatous leprosy and erythema nodosum leprosum (type 2 reaction). She was treated once a month with minocycline, moxifloxacin, and rifampin; her hand swelling improved after one month of therapy.	Gupta et al., 2021 [16]
Lymphedema	Lymphedema may be primary (from spontaneous or hereditary lymphatic architecture disorders) or secondary and attributable to numerous etiologies such as malignancy (such as breast cancer, lymphoma, and melanoma), lymph node evaluation (such as sentinel node biopsy or axillary dissection), radiation therapy, and parasitic infection (such as filariasis caused by the nematode Wuchereria bancrofti). Lymphedema-associated hand swelling initially presents as asymptomatic pitting edema; subsequently, fibrosis in the subcutaneous fat may result in non-pitting edema. Pain or sensations of heaviness and tightness may develop. Thickening of the skin can result in a peau d’orange appearance and confluent plaques of cobble-stoned, hyperkeratotic papules (elephantiasis nostra verrucosa).	Studdiford and Stonehouse, 2009 [3]
Mixed connective tissue disease	In a study of adults in Olmstead County, Minnesota residents 18 years and older diagnosed with mixed connective tissue disease between January 1, 1985, and December 31, 2014, 50 individuals were identified; hence, the annual incidence of mixed connective tissue disease was 1.9 per 100,000 population. At fulfillment of criteria, the most prevalent manifestations of this disease were arthralgias (86%), Raynaud’s phenomenon (80%), and swollen hands (64%).	Ungprasert et al., 2016 [17]
Puffy hand syndrome	Puffy hand syndrome usually presents as bilateral painless erythematous swelling of the dorsal hands often beginning several years after cessation of intravenous drug use. The hand swelling (pitting edema) initially occurs from lymphatic obstruction; subsequently, lymphatic injury and destruction result in fibrosis of the subcutaneous tissue and the edema becomes non-pitting and permanent. This condition can also be a clinical sign of diagnosed or unsuspected hepatitis C virus infection; indeed, in this setting, it has been referred to as “puffy-hand sign” and “hepatitis C hands”.	Studdiford and Stonehouse, 2009 [3], Chatterjee, 2021 [18]
Rheumatoid arthritis	A 28-year-old woman presented with persistent pain and swelling of the knuckles and wrist of three months duration. Examination showed bilateral symmetric swelling and tenderness of metacarpophalangeal and proximal interphalangeal joints of the second and third fingers. Additional evaluation established a diagnosis of seronegative rheumatoid arthritis; she was treated with prednisone (10 milligrams per day) and hydroxychloroquine sulfate (200 milligrams twice daily).	Studdiford and Stonehouse, 2009 [3], Raza et al., 2010 19]
Scleroderma	A Delphi consensus study to identify criteria for the very early diagnosis of systemic sclerosis identified three domains: skin, laboratory, and vascular. The skin domain included puffy fingers (a non-specific clinical sign of systemic sclerosis that can be seen in other diseases by would prompt a referral for systemic sclerosis) and puffy swollen digits turning into sclerodactyly (a more specific sign of systemic sclerosis indicating evolution of the condition to a fibrotic systemic sclerosis phenotype) as separate criteria items.	Avouac et al., 2011 [20]
Systemic organ disease	Heart (congestive heart failure), kidney (chronic renal disease or nephrotic syndrome) and liver (hepatic disease or failure) conditions can have accompanying edema and manifest swelling not only of the hands but also the lower extremities and/or the abdomen (in patients with anasarca).	Studdiford and Stonehouse, 2009 [3]

POTASH is a relatively common cause for asymptomatic, acquired, and spontaneously resolving, hand swelling of acute onset. The acronym has been created as a memory aid for both healthcare providers and patients to recall this etiology for hand swelling. POTASH is derived as follows: P, O, and T, are the first, second, and fourth letters of ‘post’, A is the first letter of ‘ambulatory’, S is the first letter of ‘swollen’, and H is the first letter of ‘hands’ (Table 3). Potash, on the other hand, is a water-soluble form of potassium. Primarily used in fertilizers as a nutrient to support plant growth; it not only increases both crop yield and disease resistance but also enhances water preservation. The term potash is derived from ‘pot ash’ which refers to the process for manufacturing this product by soaking either plant ash or wood ash in a water-containing pot.

**Table 3:** Derivation of POTASH acronym POTASH: post ambulatory swollen hands.

Letter	Source
P	The first letter of the word post.
O	The second letter of the word post.
T	The fourth letter of the word post.
A	The first letter of the word ambulatory.
S	The first letter of the word swollen.
H	The first letter of the word hands.

I was rather surprised to discover, to the best of my knowledge, that POTASH has rarely been described in the medical literature. Indeed, except for this paper, the only scientific publication that I am aware of on this condition was published a decade ago by a group of investigators in Brazil (Table 4) [1]. However, several sites are available on the Internet and individuals can use the term “swollen hands” to search for the diagnosis regarding why their hands swell after hiking, running, or walking [2].

**Table 4:** Summary of POTASH articles CR: current report; PAHS: postambulatory hand swelling; POTASH: post ambulatory swollen hands; Ref: reference

Author	Year	Descriptive nomenclature in article title	Syndrome and/or acronym	Comment	Ref
Ravaglia et al.	2011	Postambulatory hand swelling	Big hand syndrome and PAHS	A prospective, brief point-in-time, in-person survey (undertaken in Portuguese) of once monthly, local park, walkers. The investigators had three goals: identify the incidence of big hand syndrome in walkers, compare the prevalence of the syndrome not only in men and women but also in different age groups, and determine if dog walkers were more prone than non-dog walkers to the syndrome.	[1]
Cohen	2021	Post ambulatory swollen hands	POTASH	An autobiographical case report of running-associated POTASH, which includes a comprehensive review of post ambulatory hand swelling, a description of the positive fist sign as a clinical feature of swollen hands, and the differential diagnosis of conditions that can cause acute and chronic hand swelling.	CR

In 2011, researchers in San Paulo were initially attempting to evaluate the prevalence of not only dog ownership but also dog walking in walkers. Unexpectantly, during their pilot investigation, several of the subjects reported post ambulatory hand swelling. Therefore, the study was modified to include data on epidemiology (regarding age and gender), dogs (regarding ownership and walking), and hand swelling (regarding occurrence after walking and remitting within or persisting after 24 hours). Criteria for hand swelling included difficulty for the subject either to make a fist, or remove a ring, watch, or wrist band [1].

Unilateral or bilateral hand swelling after walking was observed in 24% (269 of 1009) of the subjects; swelling was statistically more common in people who were 3.5 years younger than their age and gender-matched counterparts. In individuals younger than 60 years old, swollen hands were observed twice as often in women than in men; after age 60 years, the prevalence was almost identical. Post walking resolution of hand swelling occurred within one to two hours in 88% (228 of 258) of the individuals; the people with persistent hand swelling after 24 hours tended to be more than five years older than those whose swelling resolved more rapidly [1].

In conclusion, the researchers observed that hand swelling occurred more commonly among dog owners (28%, 124 of 317 individuals) as compared to non-dog owners (22%, 145 of 523 individuals). However, like my observation ten years later, the investigators of this landmark study were also shocked that there was no prior description of post ambulatory hand swelling in the scientific literature. Indeed, they challenged future investigators to explore this relatively common, yet virtually totally ignored, phenomenon [1].

My POTASH developed within one hour after I had begun running. There were no associated symptoms. However, I was aware that my hands were swollen. There was swelling of both the dorsal and palmar surfaces of the hand, with loss of distinguishing features from the back of the hand such as skin lines, superficial vessels, and tendons.

The thumb and all the fingers also swell in POTASH. The digits look like sausages and accentuated swelling of the skin adjacent to rings can be observed. It also eventually becomes impossible to clench the fingers together toward the ipsilateral palm to make a fist [1,2].

A positive fist sign is defined by the inability to make a fist when the fingers are clenched. This clinical feature has been observed as an important diagnostic feature in other conditions such as acromegaly [12]. In addition to POTASH, several of the conditions that are associated with either acute or chronic hand swelling have been characterized by a positive fist sign (Table 5) [1,3,5,8,10,12,13].

**Table 5:** Conditions with a positive fist sign ^a ^A fist is created when there is clenching of the fingers and the fingertips are in direct contact with the palm of the hand with the thumb lying on top of the fingers between the proximal and distal interphalangeal joints. A positive fist sign is defined as the inability of the individual to make a fist; it, therefore, occurs when the fingertips are not able to contact the ipsilateral palm when the person attempts to clench their fingers. Therefore, a negative fist sign is observed when the individual can make an intact fist when they clench their fingers. ^b ^It is likely several, if not all, of the etiologies of acute hand swelling or chronic hand swelling are associated with a positive fist sign. However, most of the reports of patients with swollen hands do not describe whether the individual was not able to make a fist. ^c ^Resolution depends on the etiology of the hand swelling and appropriate initiation of intervention which often requires surgery such as an emergency fasciotomy. CR: current report; POTASH: post ambulatory swollen hands; Refs: references; RS3PE: remitting seronegative symmetrical synovitis with pitting edema.

Condition^a,b^	Resolves	Mechanism	Refs
Acromegaly	No	Soft tissue and bone overgrowth	Harada et al., 2019 [12]
Compartment syndrome	Post-surgery	Edema, hemorrhage, infection, and/or trauma^c^	Oak and Abrams, 2016 [5]
Giant lipoma-associated carpal tunnel syndrome	Post-surgery	Space occupying lesion and nerve compression	Jalan et al., 2011 [13]
POTASH	Spontaneously	Edema	Ravaglia, et al., 2011 [1], CR
Puffy hand syndrome	No	Lymphatic obstruction and fibrosis	Studdiford and Stonehouse, 2009 [3], Chatterjee, 2021 [18]
RS3PE	Post-corticosteroids	Edema	Joshi et al., 2009 [10]

The mechanism of pathogenesis for the positive fist sign can involve the hands, the digits, or both. The etiology may be from edema and swelling, induration and/or fibrosis with subsequent restriction of flexibility, lymphatic obstruction, and injury, soft tissue and bone overgrowth, or a space-occupying lesion with nerve compression. The hand swelling may completely resolve, as typically observed in persons with POTASH, and the individual is subsequently able to make a tight fist and thereby demonstrate a negative fist sign (Table 5) [1,3,5,8,10,12,13].

The pathogenesis of POTASH--whether it presents as unilateral or bilateral hand swelling--remains to be established. Several hypotheses have been suggested (Table 6) [1,2]. Indeed, the etiology may be multifactorial. I concur with Ravaglia et al. that additional research to determine the cause of POTASH is warranted [1].

**Table 6:** Postulated mechanisms of POTASH pathogenesis POTASH: post ambulatory swollen hands; Refs: reference

Etiology	Comments	Refs
Autonomic dysfunction	This mechanism of hand swelling is analogous to that observed in patients with complex regional pain syndrome type I (which was previously referred to as reflex sympathetic dystrophy) or following trauma to the hand or brachial plexus.	Ravaglia et al., 2011 [1]
Cold-induced vasodilation	Blood flow increases to the heart, legs, lungs, and other muscles during exercise. As this is occurring, blood flow to the hands is decreased. Subsequently, the hands become cold; in response, the vessels in the hands dilate. This causes edema in the tissues around the vessels and thereby the hands to swell.	Ravaglia et al., 2011 [1], DesMarais, 2021 [2]
Exercise-altered metabolic rates	During exercise, the muscles in the arms may not be used as much as other muscles--even if the arms are swinging back and forth. Hence, there is decreased blood flow from the arms and edema of the arms and hands occurs.	Ravaglia et al., 2011 [1]
Heat-triggered vasodilation	Exercise causes the muscles to generate heat. The body then redirects blood flow to the skin surface, at locations such as the hands, to dissipate the excess heat. The hands experience perspiration and there is vasodilation of the superficial blood vessels in the hands, resulting in hand swelling.	Ravaglia et al., 2011 [1]
Hyponatremia	This etiology of hand edema is serious and can be life threatening. It can occur in individuals who drink too much water prior to extreme and/or prolonged exercise and do not take in enough salt. This can result in a low sodium in their blood. The body tries to compensate and resolve the issue by allowing the diffusion of hypotonic fluid from the vessels into the adjacent soft tissue. Hands and feet become swollen, in addition to other areas. Hyponatremia is also associated with systemic symptoms: confusion, dizziness, headaches, irritability, muscle cramps, nausea, vomiting, and weakness.	Ravaglia et al., 2011 [1], DesMarais, 2021 [2]
Improper arm motion	Centrifugal forces result in excess fluid being mobilized into the hands.	Ravaglia et al., 2011 [1]
Reduced venous return	This mechanism of hand swelling is analogous to that observed in patients with carpel tunnel syndrome, Raynaud’s phenomenon, scleroderma, and thoracic outlet syndrome. It also occurs from gravity-associated blood pooling; if the arms are at the person’s sides, gravity pools blood into the hands and causes swelling. This etiology might be exacerbated in hikers who wear a tight-fitting backpack.	Ravaglia et al., 2011 [1], DesMarais, 2021 [2]
Systemic neurogenic effect	Unexplained swelling of the hands was observed in a patient following acupuncture treatment.	Ravaglia et al., 2011 [1]

Based on my personal experience, POTASH is an acquired condition. I have participated in competitive running since age 13 years (ninth grade) on the track and field team; in college, I was a member of both the cross country and the track and field teams. Daily running practice was rarely more than 1½ hours and races were six to eight miles (10 to 13 kilometers). I never experienced any hand swelling.

I began marathon running in 1990 in Houston, Texas; I subsequently completed 20 Houston marathons during the next 25 years. The races occurred in mid-January; temperatures at the start of the race ranged from upper 30 degrees Fahrenheit to lower 50 degrees Fahrenheit and would increase by 10 to 15 degrees by the end of the race. Most of my races were completed within four to 4½ hours. I did not develop hand swelling during any of the marathons.

I developed POTASH in 2016. Since the initial episode, I have noted that the clinical recurrence of POTASH usually happens when I run for more than 60 minutes; I have not experienced the hand swelling to develop during any other activities. However, the degree of swelling is proportional to the running time duration; less swelling of the hands occurs after a shorter running time than that which has been observed after nearly four hours of running. 

My episodes of POTASH were not related to the running location--either with regards to the elevation above sea level or the temperature. Although the race distance I now run has decreased to 13.1 miles, the running time has increased to between three hours and 15 minutes to three hours and 45 minutes. POTASH has occurred during half marathons I have run in Houston (Texas), Northbend (Washington), and San Diego (California). The ambient temperature during the running event has varied from the upper 30 degrees Fahrenheit to the mid 70 degrees (or even warmer).

Can POTASH occur in people other than those individuals who ambulate outside such as hikers, long-distance runners, and walkers? POTASH is an often-overlooked condition and is likely to be not only under-diagnosed but also under-reported. Hence, objective data to answer this question remains to be determined. The critical features for the development of POTASH include both an ambulatory activity and sustaining that activity for an adequate duration of time to elicit hand swelling; the actual distance ambulated may--or may not--be a contributory factor. Therefore, it is indeed possible that POTASH could be observed not only in athletes such as soccer players during long practices or games but also in fitness enthusiasts who have prolonged workouts, often indoors, that consist of exercising by either walking on a treadmill or running on an elliptical trainer.

The treatment of POTASH is observation and reassurance. Once the inciting event--be it hiking, running, or walking--is discontinued, the hand swelling spontaneously resolves. The swelling of my hands disappeared within two hours. Similarly, Ravaglia et al. noted that hand swelling completely cleared within one to hours for 88% of their patients [1].

Can POTASH be prevented? Ravaglia et al. noted that dog owners who regularly walked their dogs had a reduced prevalence of post ambulatory hand swelling as compared to owners who did not maintain a regular schedule for dog walking. Therefore, based on their observation, if you are a dog owner and if you walk your dog, POTASH is less likely to occur if you walk your dog regularly [1].

If you are a hiker or a walker or a dog owner (but do not regularly walk the dog) who has experienced POTASH, it might be prudent to remove rings from your fingers prior to going on the hike or walk; similarly, removal of a bracelet, a fitness tracker, or a watch may be considered. In addition, several suggestions have been proposed to minimize or prevent POTASH (Table 7) [2]. In addition to isotonic hydration and electrolyte-containing snacks, periodic and purposeful upper extremity movement and pressure relief during the hike or walk and wearing not only non-constrictive clothing but also a backpack with tension-free shoulder straps are additional techniques that may help to eliminate the development of swollen hands after hiding or walking [2].

**Table 7:** Techniques that may prevent hiking-related or walking-associated POTASH

Technique	Comments
Accessory (backpack and clothing) considerations	Loose, non-constricting clothing should be worn to allow unrestricted blood flow. Bra straps and shirt sleeves that are tight-fitting should be avoided. If a backpack is being worn, the shoulder straps should be adjusted so that the pack is tension-free and comfortably lies on the back.
Arm engagement	Make a conscious effort that the arms are moving. If the hands are kept on the side of the body or holding onto the backpack strap, there is minimal movement of the arms. Hiking poles increase movement and bending of not only the arms, but also the fingers, hands, and shoulders; therefore, using hiking poles may prevent hand swelling by continually engaging the upper extremity.
Electrolyte replacement	Isotonic drinks that contain sodium (www.gatorade.com) is a safe approach to liquid rehydration during hiking and walking. Electrolyte drink tablets that dissolve in water (www.nuunlife.com) can also be used. Electrolyte replacement can also be achieved with supplements such as gel packs and gummies (www.saltstick.com).
Glove wearing	Snugly fitting, but not too tight, compression gloves may reduce hand swelling--especially in for those individuals with recurrent hiking-related or walking-associated POTASH. Depending on the season and ambient temperature, either a light pair of gloves with moisture-wicking fabric (for the summer and warmer climates) or a heavier pair of gloves (for the winter and colder climates) should be considered.
Hand exercises	These may help to avoid hand swelling; in addition, if swollen hands have developed, they may aid in expediting the resolution of the swelling. Initially raising the arms above the head; then either massage each finger (from the fingertip to the hand) or wiggle and clench the finger or both.
Pressure relief intervention	If a heavy backpack is being carried, constricting pressure from the straps can cause swelling of the underlying shoulder and arms. This can be relieved by placing the thumb of each hand beneath the ipsilateral strap of the backpack, lifting the pack off of the shoulders, and keeping the pack elevated from the back until the swelling has gone down.

Compression gloves have also been suggested to aid in the reduction of hike-related or walking-associated hand swelling. I did not wear gloves during an entire running event prior to my development of running-associated hand swelling. Currently, I do not continuously wear gloves when running. Therefore, I cannot assess the possible benefit of compressive gloves to prevent POTASH associated with my running.

Occasionally, prior to running and during the first-to-second mile I may wear a sock over each hand until they are warm. However, once warm hands are achieved, the socks are removed and rarely applied again during the remainder of the run. In recent years, this brief duration of hand protection from cold--using non-compressive hand clothing (such as loose-fitting mittens or gloves)--has not deterred the occurrence of POTASH.

## Conclusions

POTASH is an acquired condition characterized by acute hand swelling. The swollen hands begin to occur after starting an ambulatory activity such as hiking, running, or walking. Prior to this report, POTASH has only been described once in the medical literature a decade ago; however, hikers and walkers were aware of the potential development of ambulatory activity-associated hand swelling. From the author’s personal experience, the dorsal and palmar hand swelling is asymptomatic, bilateral, and symmetric; both hands usually being to swell after approximately one hour of running; the extent of swelling is proportional to the duration of exercise. Evaluation to assess for the fist sign is a simple test to assess not only for POTASH, but also for other causes of either acute or chronic hand swelling. Like the author, a positive fist sign--demonstrated by the inability to clench the fingers tightly into a fist--is always observed in patients with POTASH. Within two hours, the hand swelling of most patients with POTASH spontaneously resolves and a negative fist sign is noted--a fist of tightly clenched fingers can be formed. Recommendations have been provided to potentially eliminate or limit the degree of POTASH. Although several possible mechanisms of pathogenesis have been suggested, the definitive etiology of POTASH remains to be established and additional investigation to determine the cause of ambulatory-related swollen hands is warranted.
